# Supplementary material for: The Optimal Cutoff Value of Tumor Markers for Prognosis Prediction in Ampullary Cancer
Source: Cancers (Basel). 2023 Apr 13;15(8):2281. doi: 10.3390/cancers15082281 (PMC10136701; doi:10.3390/cancers15082281)
Supplement: Supplementary file 1 [file cancers-15-02281-s001.zip › Supplement Figure S1.pdf]

(a)

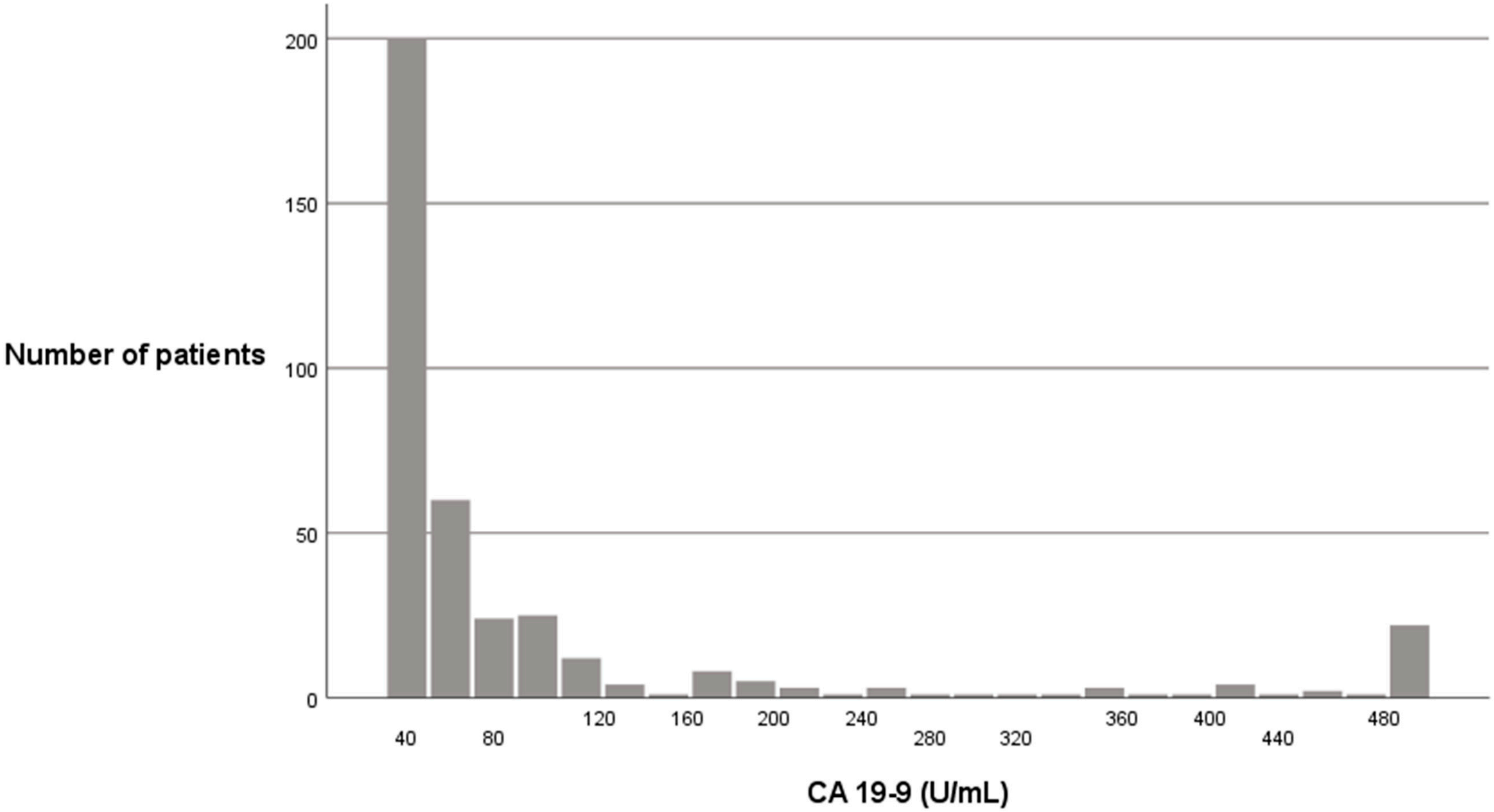

Number of patients
200
150
100
50
0
40
80
120
160
200
240
280
320
360
400
440
480
CA 19-9 (U/mL)

(b)

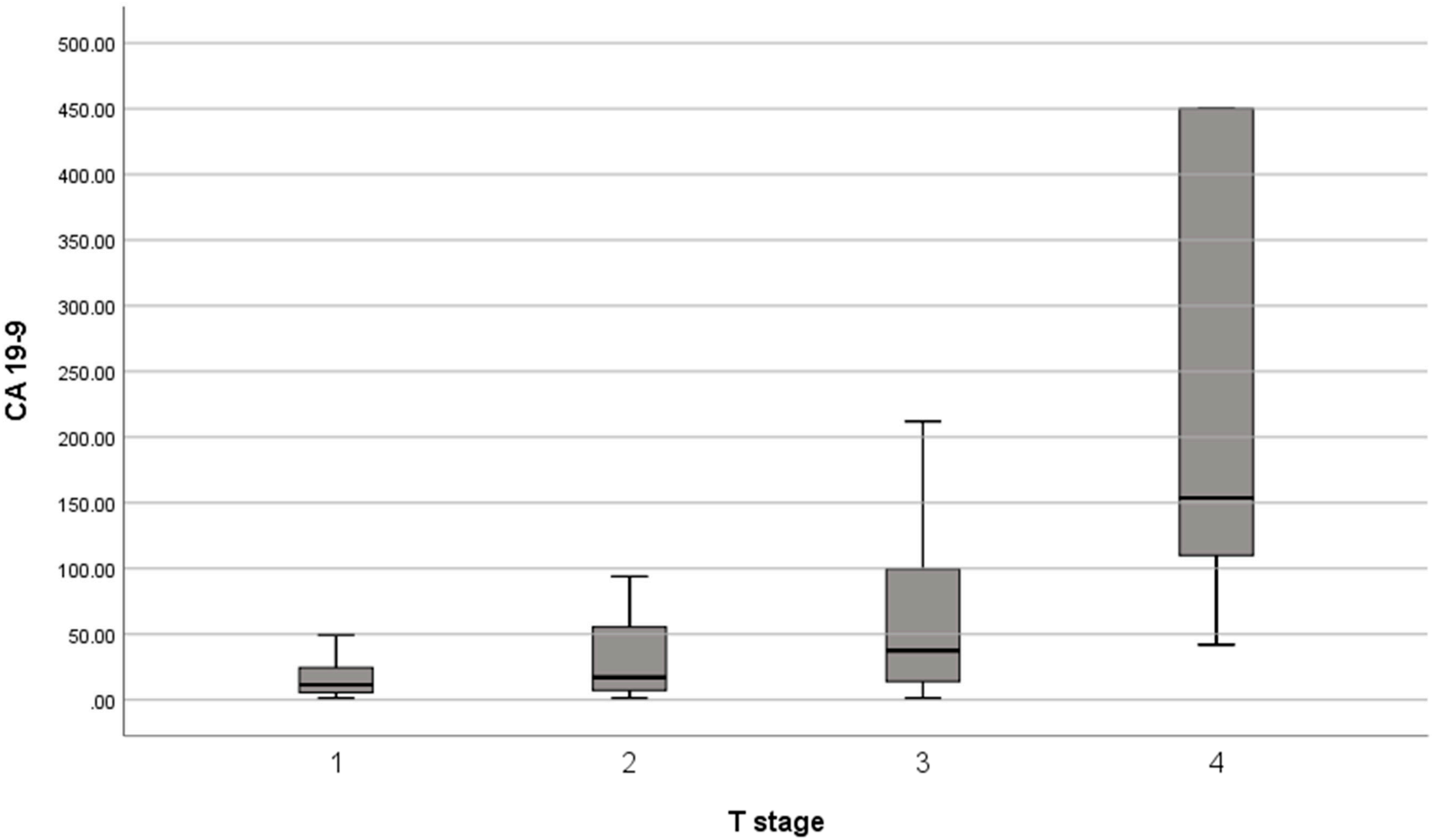
CA 19-9
500.00
450.00
400.00
350.00
300.00
250.00
200.00
150.00
100.00
50.00
.00
1
2
3
4
T stage

(c)

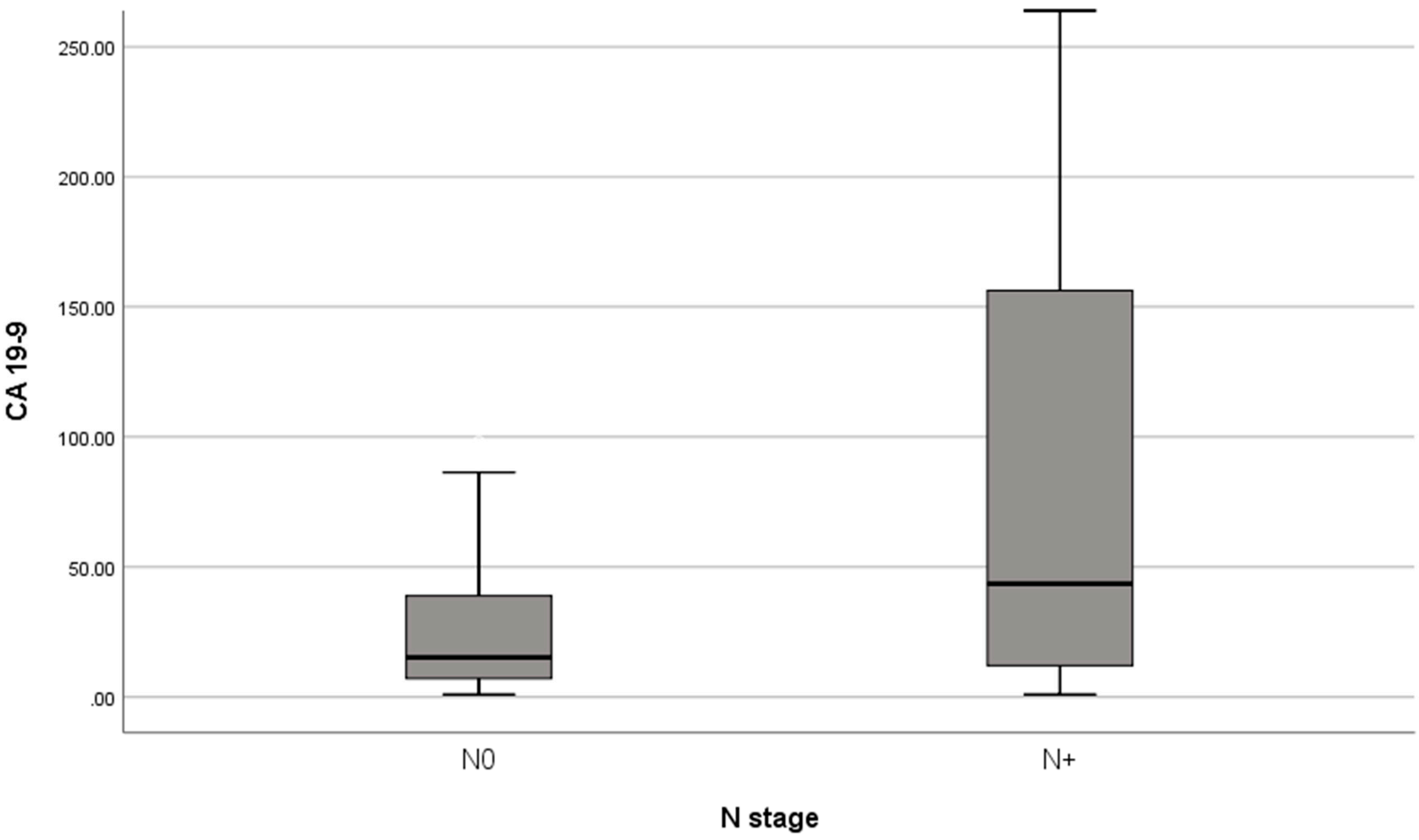

Supplement Figure S1. (a) Tumor marker distribution **(b)** Tumor marker distribution in T stage **(c)** Tumor marker distribution in N stage
