# Supplementary material for: The Optimal Cutoff Value of Tumor Markers for Prognosis Prediction in Ampullary Cancer
Source: Cancers (Basel). 2023 Apr 13;15(8):2281. doi: 10.3390/cancers15082281 (PMC10136701; doi:10.3390/cancers15082281)
Supplement: Supplementary file 1 [file cancers-15-02281-s001.zip › Supplement Figure S2.pdf]

(a)

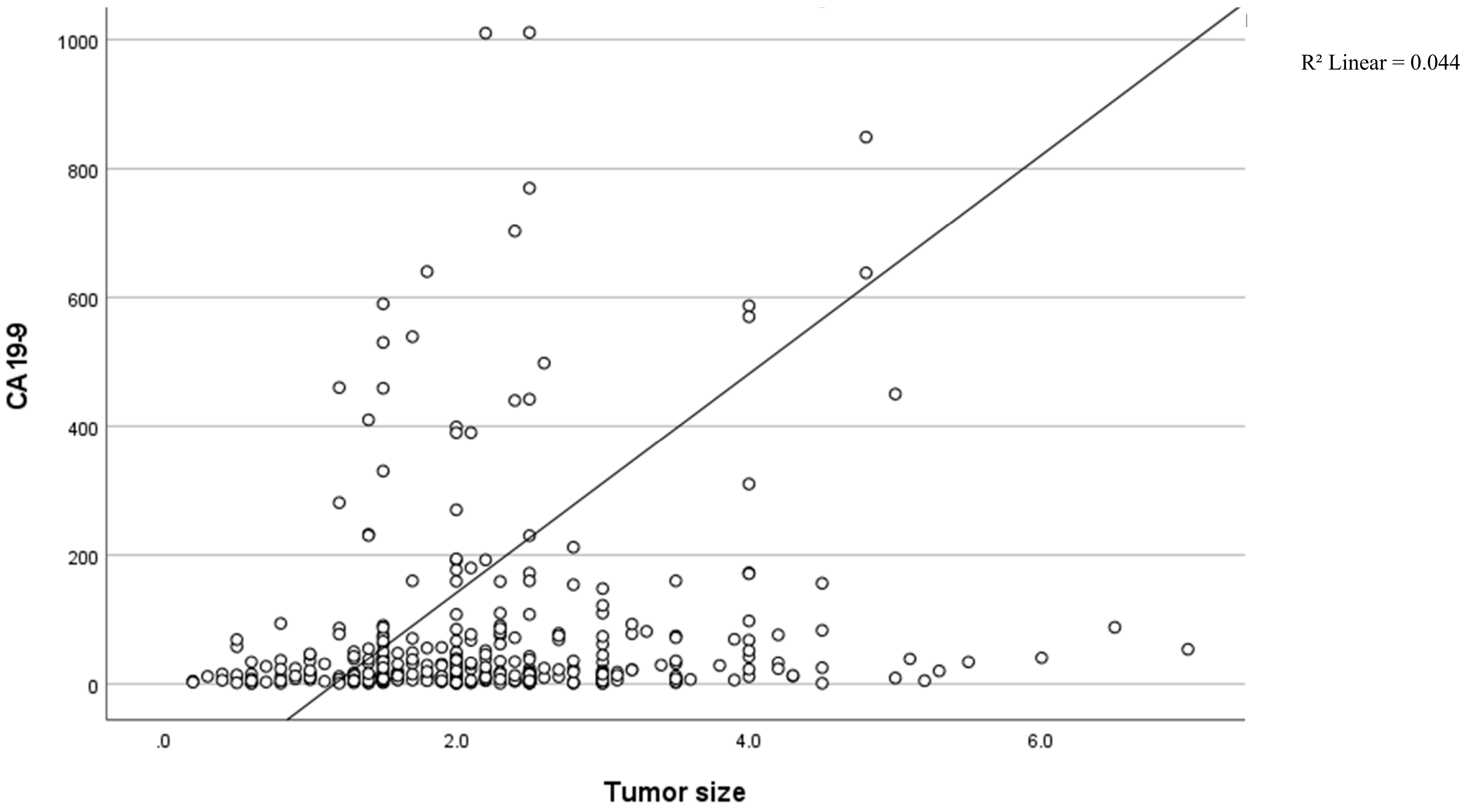

CA 19-9
1000
800
600
400
200
0
.0
2.0
4.0
6.0
Tumor size
R² Linear = 0.044

(b)

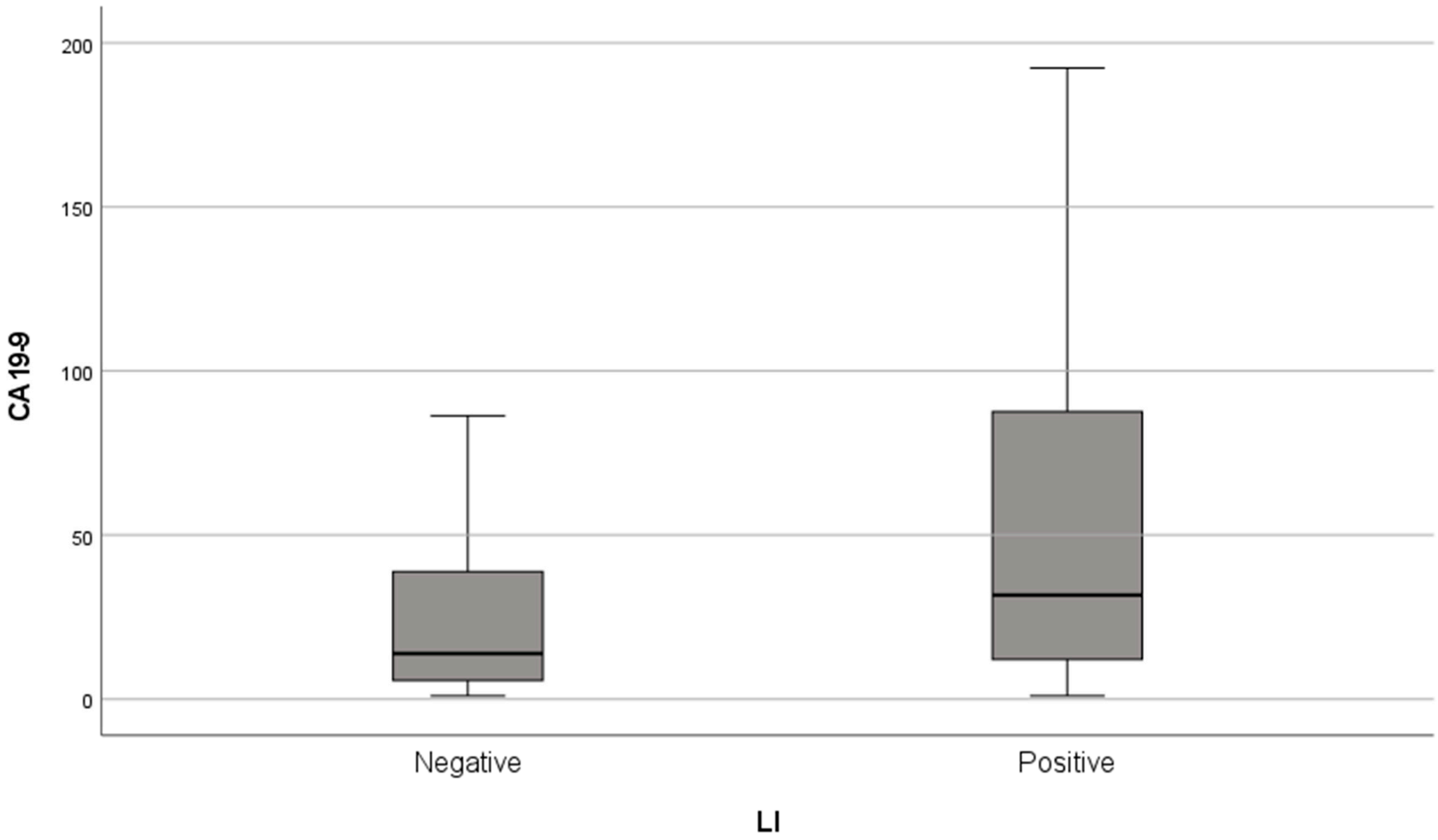

(c)

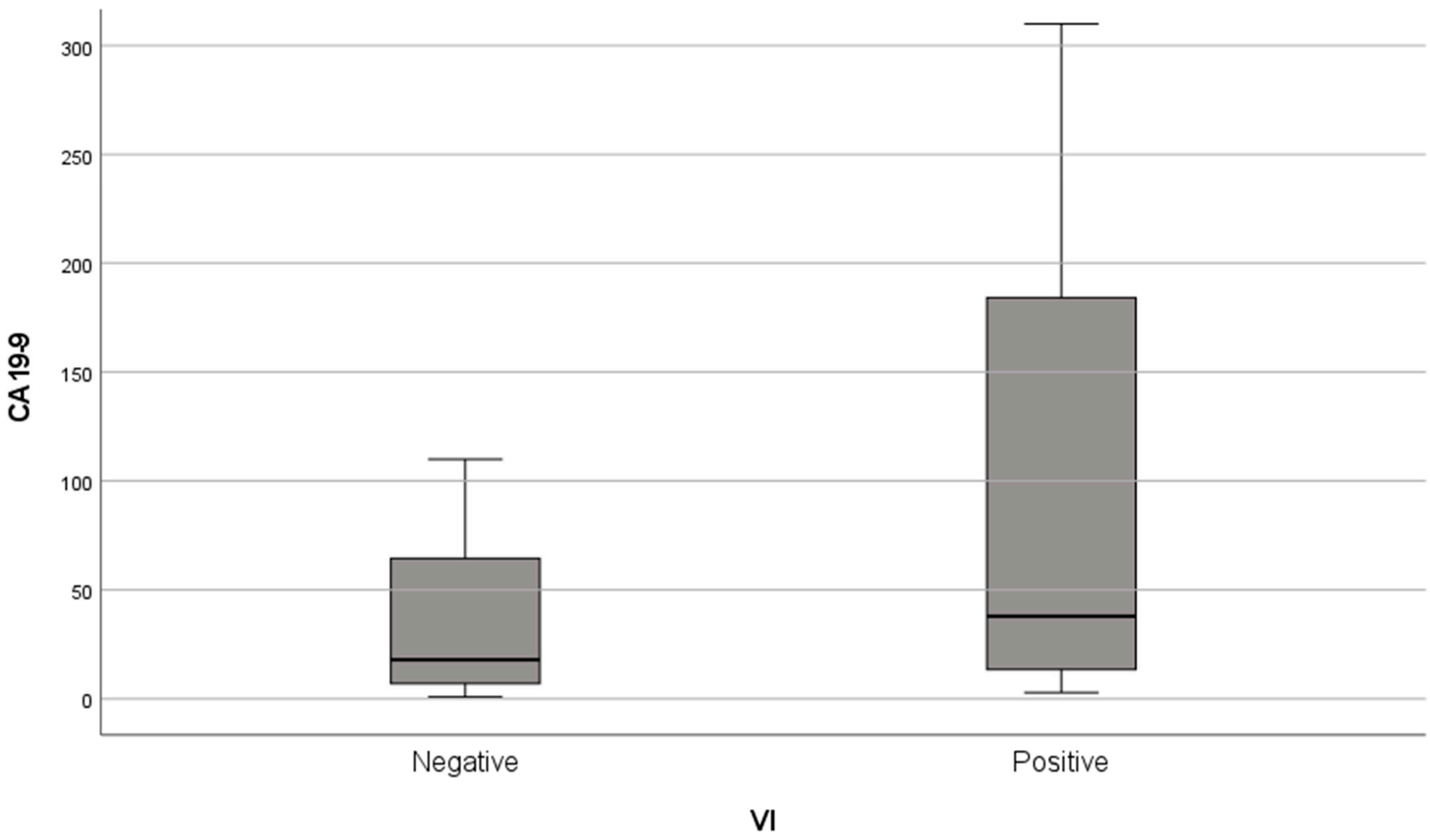

(d)

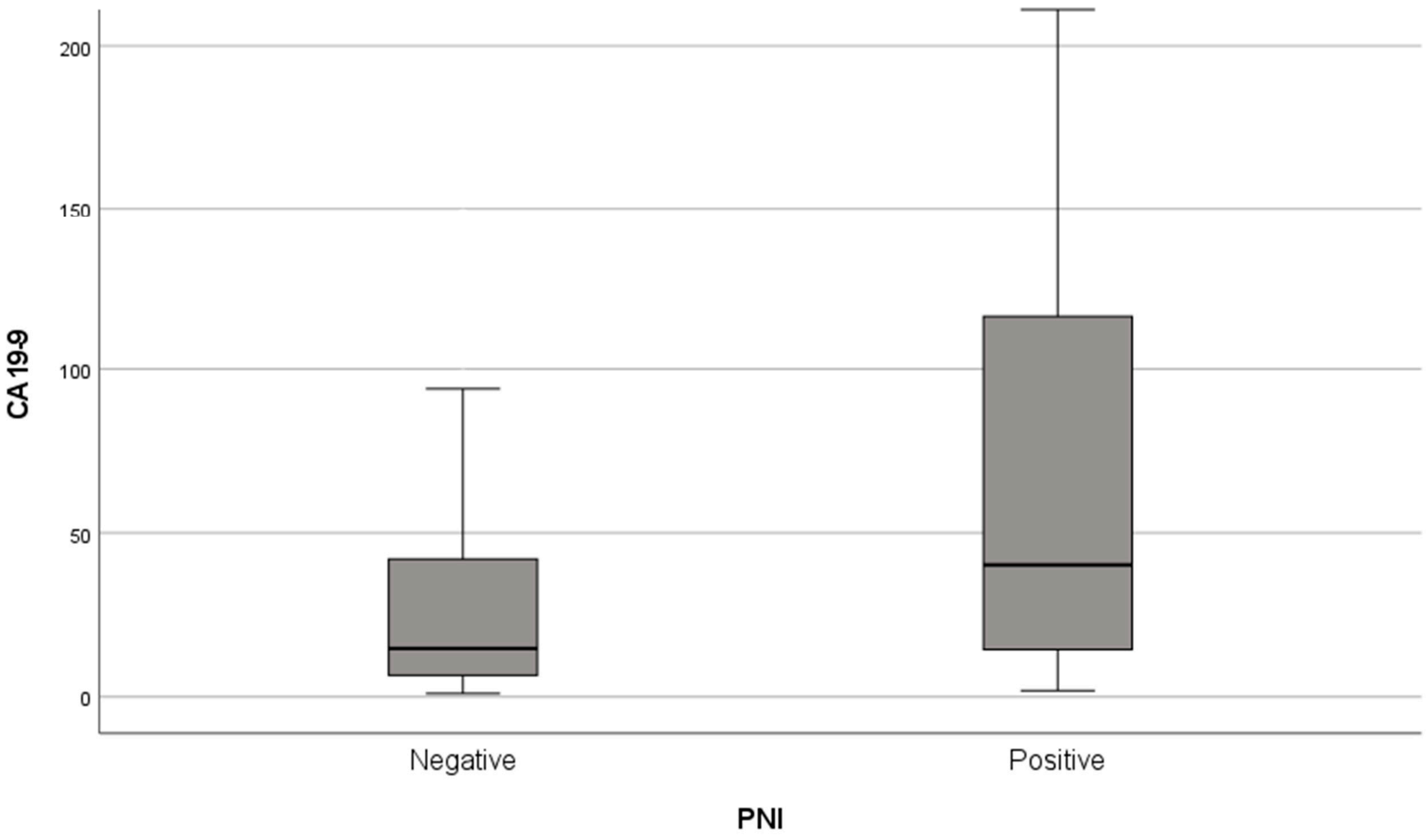

Supplement Figure S2. (a) CA 19-9 level according to tumor size **(b)** CA 19-9 level according to lymphatic invasion (LI) **(c)** CA 19-9 level according to venous invasion (VI) **(d)** CA 19-9 level according to perineural invasion (PNI)
