# Supplementary material for: The Optimal Cutoff Value of Tumor Markers for Prognosis Prediction in Ampullary Cancer
Source: Cancers (Basel). 2023 Apr 13;15(8):2281. doi: 10.3390/cancers15082281 (PMC10136701; doi:10.3390/cancers15082281)
Supplement: Supplementary file 1 [file cancers-15-02281-s001.zip › Supplement Figure S3.pdf]

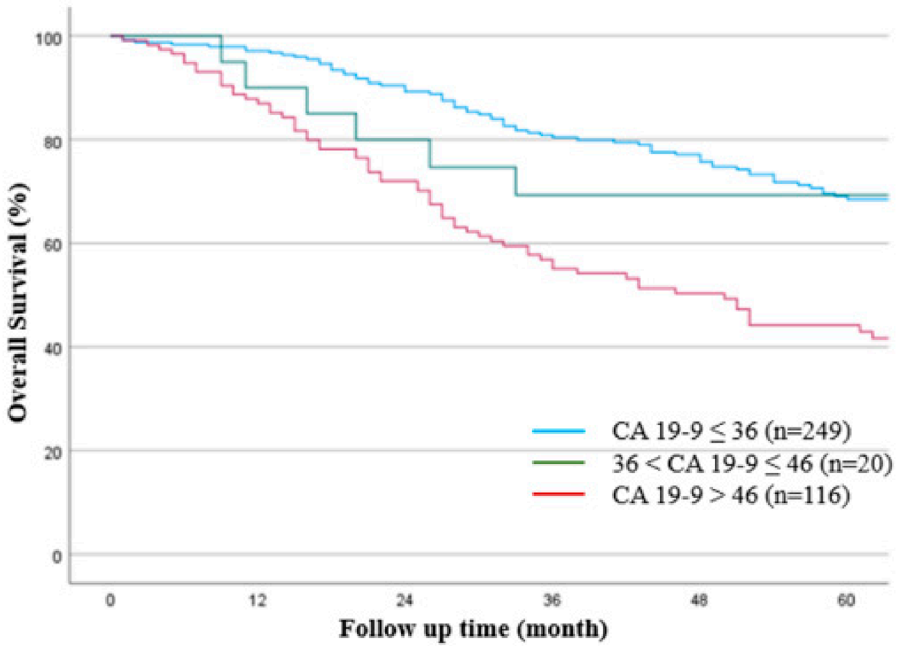

Risk group	5yr OS rate (%)	Median (m)
CA 19-9 ≤ 36	69.1	Not reached
36 < CA 19-9 ≤ 46	69.3	Not reached
CA 19-9 > 46	44.2	50.0

Supplement Figure S3. Survival outcomes according to the CA 19-9 level subgroups
