# Supplementary material for: The Optimal Cutoff Value of Tumor Markers for Prognosis Prediction in Ampullary Cancer
Source: Cancers (Basel). 2023 Apr 13;15(8):2281. doi: 10.3390/cancers15082281 (PMC10136701; doi:10.3390/cancers15082281)
Supplement: Supplementary file 1 [file cancers-15-02281-s001.zip › Supplement Table S1.pdf]

Supplement Table S1. Prognostic factors for overall survival (CA 19-9 level cut-off: 36 U/mL).

			Univariate analysis		Multivariate analysis	
		Patients (n=385)	HR (95% CI)	P-value	HR (95% CI)	P-value
Preoperative CEA , ≤5.0/>5.0 (ng/mL)		362/23	1.80 (1.07-3.01)	0.026	1.11 (0.65-1.88)	0.523
Preoperative CA19-9 , ≤36/>36 (U/mL)		249/136	1.90 (1.41-2.55)	<0.001	1.25 (0.91-1.71)	0.123
Total bilirubin ,<2.0/≥2.0 (mg/dl)		215/170	1.54 (1.14-2.09)	0.005	1.38 (1.01-1.88)	0.044
Histologic grade	WD	128	Reference	-	Reference	-
	MD	210	2.29 (1.56-3.35)	<0.001	1.84 (1.25-2.72)	0.002
	PD	34	5.77 (3.45-9.64)	<0.001	4.77 (2.82-8.06)	<0.001
T stage	T1	128	Reference	-	Reference	-
	T2	119	1.87 (1.21-2.90)	0.005	1.00 (0.63-1.60)	0.176
	T3/4	138	3.14 (2.08-4.73)	<0.001	1.36 (0.85-2.16)	0.042
N stage	N0	271	Reference	-	Reference	-
	N+	114	2.82 (2.09-3.79)	<0.001	1.75 (1.27-2.43)	0.001
R status	R0	383	Reference	-	Reference	-
	R1	2	1.16 (0.16-8.30)	0.881	0.70 (0.10-5.11)	0.767
Adjuvant chemotherapy	No	203	Reference	-	Reference	-
	Yes	182	2.84 (2.07-3.90)	<0.001	2.19 (1.55-3.09)	<0.001
